# Information Transmission in a Neuron-Astrocyte Coupled Model

**DOI:** 10.1371/journal.pone.0080324

**Published:** 2013-11-29

**Authors:** Jun Tang, Jin-Ming Luo, Jun Ma

**Affiliations:** 1 College of Science, China University of Mining and Technology, Xuzhou, China; 2 Department of Physics, Lanzhou University of Technology, Lanzhou, China; University of Maribor, Slovenia

## Abstract

A coupled model containing two neurons and one astrocyte is constructed by integrating Hodgkin-Huxley neuronal model and Li-Rinzel calcium model. Based on this hybrid model, information transmission between neurons is studied numerically. Our results show that when the successive spikes are produced in neuron 1 (N1), the bursting-like spikes (BLSs) occur in two neurons simultaneously during the spikes being transferred to neuron 2 (N2). The existence of the astrocyte and a higher expression level of mGluRs facilitate the occurrence of BLSs, but the rate of occurrence is not sensitive to the parameters. Furthermore, time delay *τ* occurs during the information transmission, and *τ* is almost independent of the effect of the astrocyte. Additionally, we found that low coupling strength may result in the distortion of the information, and this distortion is also proven to be almost independent of the astrocyte.

## Introduction

Although the number of the glial cells is several times larger than that of the neurons in most parts of the brain, few studies have focused on the effect of glial cells on neuronal behavior. Over the past decades, an increasing number of works have demonstrated that the interaction between glial cell and neuron serves an important function in information transmission in the neuron system [1]–[3]. Astrocytes are the most numerous type and the best studied glial cells. Astrocytes modulate synaptic transmission through many different pathways [4], [5]. The most discussed one is that the presynaptic neuron release a kind of neurotransmitter, glutamate, which activates glutamate ionotropic receptors (i-GluRs) on the postsynaptic membrane. Astrocytes participate in this synaptic transmission by responding to the glutamate in the synaptic cleft through calcium elevation; this elevation of Ca^2+^ above a certain threshold triggers the release of glutamate to the synaptic cleft [6]–[9]. Calcium elevation also results in the release of other transmitters such as Adenosine Triphosphate (ATP). ATP activates the purinergic ionotropic receptors, which facilitates the enhancement of neuronal excitability [10]–[15]. Moreover, astrocytes absorb excess potassium released by neurons in the synaptic space and thus regulate excitation [16], [17]. This bidirectional coupling between neurons and astrocytes indicates the concept of the “tripartite synapse” [18]–[21].

Traditional modeling studies of neuron system consider the coupling between the neurons, but ignore the participation of glial cells [22]–[25]. Nadkarni and Jung introduced a model accounting for the interaction between the neurons and the astrocytes [26]. They model the effect of astrocyte on neuron through a calcium-dependent inward current in the neuron. The calcium-dependent function is fitted from experimental data [27]. This kind of modeling scheme is extensively employed by many researchers [14], [15], [20], [28], [29]. The model proposed by Nadkarni and Jung predicted the seizure-like spontaneous oscillations in the absence of stimuli. Following Nadkarni and Jung, many modeling studies focus on the contribution of astrocytes to epilepsy [30]–[34] motivated by experimental findings [35]. For example, Amiri *et al.* concluded that disruption of the homeostatic function of astrocytes may initiate the hypersynchronous firing of neurons through successive research works [32]–[34]. This finding suggests that the neuron-astrocyte interaction may represent a novel target to develop effective therapeutic strategies for epilepsy.

Neurons are widely accepted to be organized into networks, and neuronal networks exchange information through electrical and chemical synapses. Increasing evidences indicate that astrocytes are also organized into networks [4], and astrocyte networks are interconnected through gap junction channels. The channels are regulated by extra- and intracellular signals that enable the exchange of information. Based on these two networks, a recent review paper suggests the concept of “astroglial networks” [2]. Many recent modeling works focus on the neuronal synchronization in the astroglial network [28], [36]–[40]. As an example, by integrating Norris-Lecar neuron model and Li-Rinzel calcium model, Amiri *et* *al.* constructed a model to study how astrocytes participate in the interplay between the pyramidal cells and interneurons [40]. Furthermore, they extended their three-unit model to a neuronal population model to study the effect of astrocyte on neuronal synchronization. Astrocytes are concluded to be capable of changing the threshold value of transition from synchronous to asynchronous behavior among neurons [39].

Postnov *et al.* proposed a model containing three units (the presynaptic, and postsynaptic neurons and the glial cell) [5]. Their model can predict the long-term potentiation of the postsynaptic neuron. In the modeling study of astrocyte-neuron interaction, pyramidal cells and interneurons are often the focus [5], [15], [39], [40]. Our study is likewise based the same coupled neurons. We will focus on the effect of astrocyte when information is transferred from pyramidal cell to interneurons, i.e., how the existence of astrocyte changes the response of interneuron to the firing pattern of pyramidal cell.

## Models

**Figure 1:**
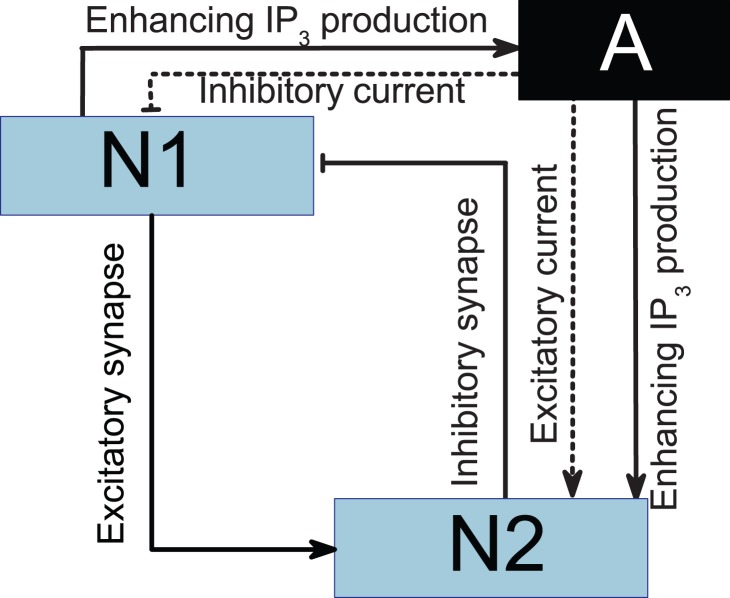
Schematic of the three-unit model. N1: pyramidal cell; N2: interneuron; A: astrocyte.

Our model, which is schematized in Fig. 1, contains two conductance-based neurons and one astrocyte. Pyramidal cells are known to excite interneurons. By contrast, the interneurons inhibit the pyramidal cells. Thus, the two neurons in the model are coupled by excitatory and inhibitory synapses. The Hodgkin-Huxley equations have served a vital function in the theoretical understanding of neuronal behavior [41]. Following Ref. [26], we use the Hodgkin-Huxley equations to model the two neurons. The model equation describing the transmembrane potential contains sodium, potassium, and leak currents. The equations are given by
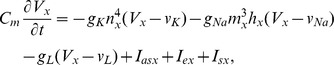


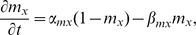


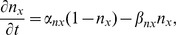


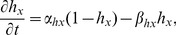
(1)where 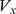 denotes the transmembrane potential of *x*th neuron (*x* = 1,2), and 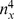 represents the fraction of open Na^+^ channels, and 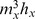 represents the fraction of open potassium channels. The values of parameters are listed in Table 1. The closing and opening rates of the gates are given by
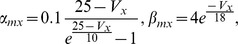


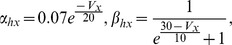


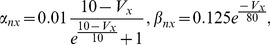
(2)where 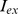 denotes the injected current input in *x*th neuron, 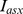 is the feedback current received from the astrocyte by *x*th neuron, and 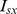 is the synaptic current received by *x*th neuron. Terman *et al.* [42] suggest that the neuron releases a neurotransmitter to the synaptic cleft depending on the membrane potential, and the concentration of neurotransmitter released by *x*th neuron is given by

**Table 1:** Parameter values.

parameter	value
*C_m_*	1 µF/cm^2^
*g_K_*	36.0 mS/cm^2^
*g_Na_*	120.0 mS/cm^2^
*g_L_*	0.3 mS/cm^2^
*v_K_*	−12.0 mV
*v_Na_*	115 mV
*v_L_*	10.6 mV
*θ_s_*	85.0
*σ_s_*	2.0
*α_s_*	0.1
*β_s_*	0.05
*g_si_*	0.1
*v_si_*	0.0 mV
*v_se_*	−85.0 mV
*c*_0_	2.0 µM
*c*_1_	0.185
*v_a_*	6 s^−1^
*v_b_*	0.11 s^−1^
*v_c_*	0.0 µM/s
*d*_1_	0.13 µM
*d*_2_	1.049 µM
*d*_3_	0.9434 µM
*d*_5_	0.08234 µM
*a*_2_	0.2 µM^−1^s^−1^
*k*_3_	0.1 µM
*P*_0_	160.0 nM
*τ_P_*	0.00014 ms^−1^
*g_se_*	variable
*r_p_*	variable

The parameter values are obtained from references with slight modification.



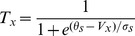
(3)Following Terman *et al.* [42], the synaptic variable 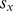 is introduced to explain the effect of the neurotransmitter release 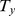 by *y*th neuron on the *x*th neuron, and the dynamic equation is given by
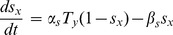
(4)


Then, the synaptic current 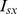 received from each other by the two neurons in our model is
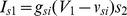


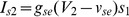
(5)where 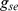 and 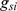 are the maximal conductance of the excitatory and inhibitory synapses. 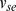 and 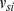 are the corresponding reversal potential.

Astrocytes do not generate action potentials, i.e., the astrocytes are non-excitable electrically. The astrocytes respond to the neurotransmitter release in the synaptic cleft through IP_3_ production[see Fig. 1]. Subsequently, elevation of IP_3_ concentration induces the release of Ca^2+^ from endoplasmic reticulum (ER), and then more Ca^2+^ are released depending on the IP_3_-induced Ca^2+^ elevation. The elevation of Ca^2+^ above a certain threshold triggers the release of glial transmitters, which, in turn, will influence the dynamics of the neurons. We use Li–Rinzel model to describe the Ca^2+^ exchange in the astrocyte[43]. This process contains three fluxes across the ER membrane: flux release through the ion channels (IP_3_Rs), removal of Ca^2+^ by an ATP-dependent pump, and a leak.
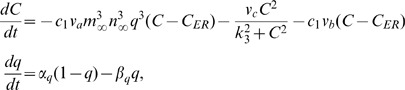
(6)


with
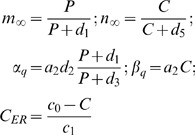
(7)


where *C* denotes the Ca^2+^ concentration in the intracellular space, *q* is the fraction of activated IP_3_R, and *P* is the IP_3_ concentration in the intracellular space. The values of parameters are listed in Table 1. The production of intracellular IP_3_ is modeled by
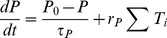
(8)


Nadkarni and Jung fit the experimental data[27] using the function of the current versus astrocytic Ca^2+^ concentration
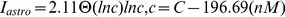
(9)


Numerous physiological studies show that astrocytes release ATP, which has direct excitatory effects on hippocampal interneurons [44], [45]. By contrast, astrocytes decrease pyramidal neuron excitability (Fig. 1) [36], [46]. These findings suggest the following current 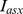 in Equation (1):
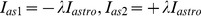
(10)where we introduce parameter 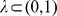 to account for the effect of astrocytes. Given that 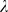 = 0, the effect of astrocyte is ignored, whereas when 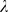 = 1, the effect of astrocyte is considered fully.

All parameter values are listed in Table 1. We solve the model Equations (1) to (10) by using a fourth-order Runge-Kutta integration scheme with a time step 0.05, and simulations verify that further time step reduction does not significantly improve accuracy.

## Results and Discussion

**Figure 2:**
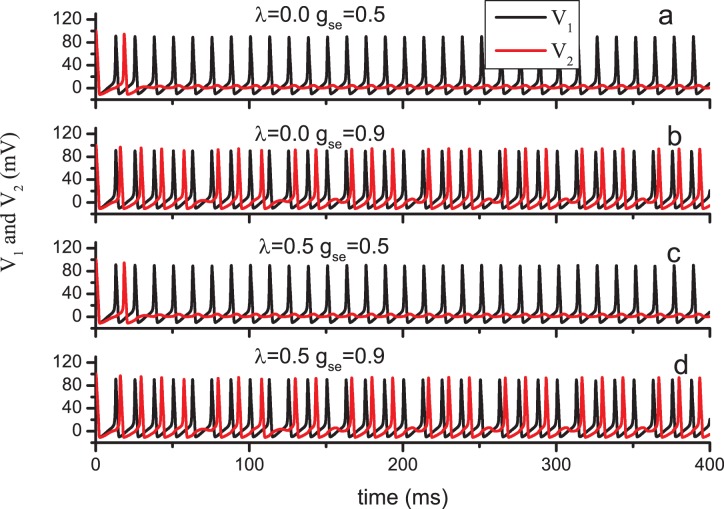
Time series of membrane potential in N1 and N2 for different parameter values. The successive spikes in N1 are induced by the injected current 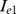 = 10.0 *µ*A/cm^2^, and 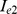 = 0.0 *µ*A/cm^2^ by which the spikes can not be induced in N2; 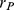 = 0.8 *µ*M/s. (a)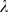 = 0, 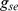 = 0.5; (b)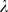 = 0, 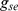 = 0.9; (c)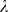 = 0.5, 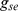 = 0.5; (d)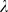 = 0.5, 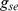 = 0.9.

**Figure 3:**
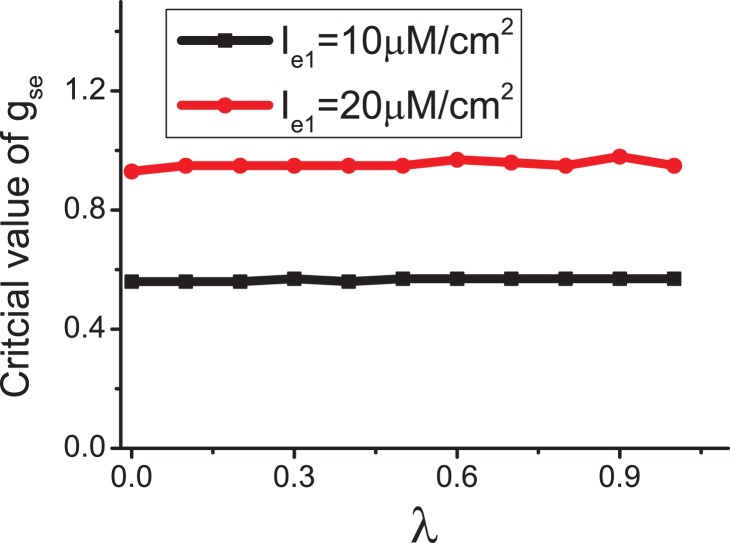
The critical value of 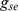 for which the information is transferred from N1 to N2. 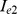
 = 0.0 *µ*A/cm^2^ by which the spikes can not be induced in N2; 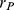 = 0.8 *µ*M/s.

Ignoring the effects of astrocyte and synaptic current, i.e., 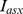 = 0 and 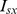 = 0, 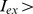
6.24 *µ*A/cm^2^ is needed to generate persistent action potentials in the isolated H–H neuron. To study the information transmission from N1 to N2, we let 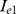 = 10.0 *µ*A/cm^2^, and 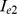 = 0.0 *µ*A/cm^2^, for which the persistent action potentials are generated in N1, but cannot be generated in N2 on its own. While the effect of the astrocyte is ignored (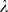 = 0), the persistent action potentials are found in N2 for large coupling strength 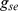, that means the information implied in the action potentials of N1 are transferred to N2. Comparing Figs. 2 (a) and (b), 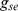 = 0.9 is sufficient for the information transmission, but 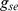 = 0.5 is not. The results of calculation show that the critical value of 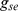 for the information transmission is 0.56. When the effect of the astrocyte is considered, i.e., 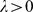, the results are not significantly changed by the astrocyte[Figs. 2(c) and (d)]. As an example, in Fig. 3, the critical values are calculated for different values of 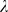. Both for 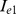 = 10.0 and 20 *µ*A/cm^2^, the critical value is independent of 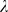 but varies with different values of 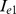.

### Bursting-like Spikes

**Figure 4:**
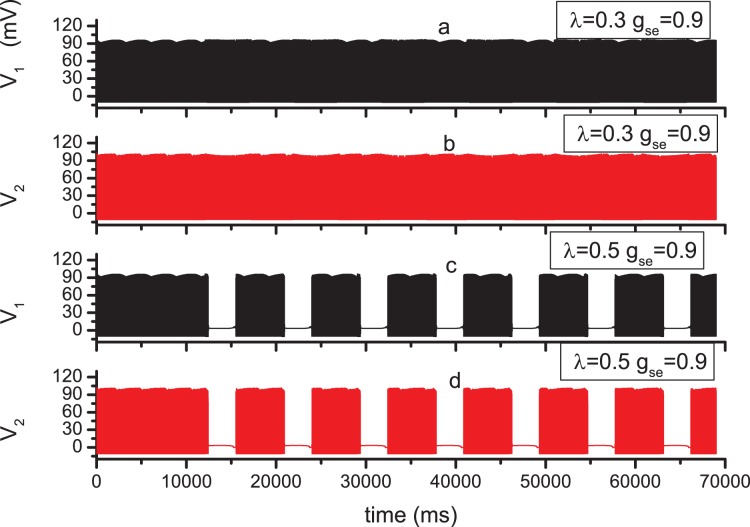
Time series of membrane potential in N1 and N2 for different parameter values. The values of parameters 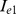, 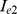 and 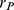 are same as in Fig. 2. (a)(b)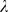 = 0.3, 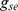 = 0.9; (c)(d)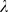 = 0.5, 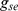 = 0.9. Note that the time scales are much longer than that in Fig. 2.

**Figure 5:**
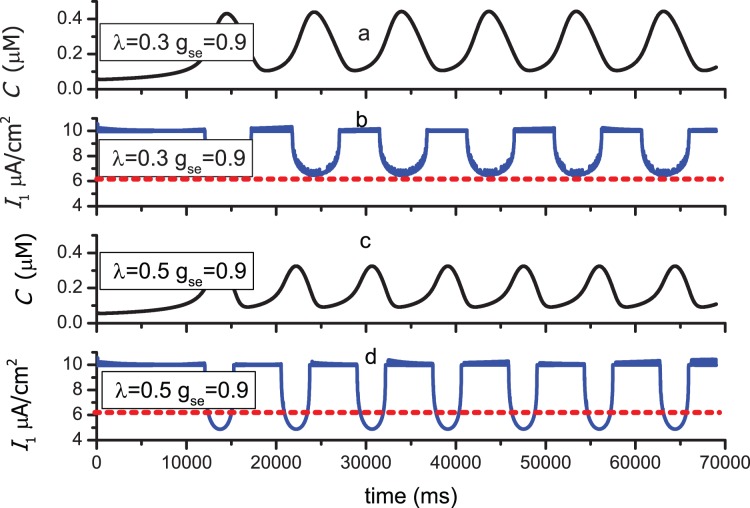
Time series of calcium concentration and total current in N1 corresponding to that of membrane potential in Fig. 4. The values of parameters ***I**_e_*_1_, ***I**_e_*_2_and ***r**_P_* are same as in Fig. 2. (a)(b) *λ* =  0.3, ***g**_se_* =  0.9; (c)(d) *λ* =  0.5, ***g**_se_*  =  0.9. The red dashed lines indicate the value 6.24 *µ*A/cm^2^.

We now focus on a longer time scale. In Fig. 4, the value of 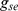 is 0.9, for which the persistent action potentials in N1 are successfully transferred to N2. When 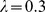, the successive action potentials are transferred. Notably, bursting-like action potentials are found both in the N1 and N2 for 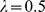. Bursting-like spikes (BLSs) are extensively found in experimental and modeling studies. Cressman Jr. *et al*. have studied the influence of sodium and potassium dynamics on neuronal behaviors using a single neuron model containing the effect of the glial cell [16], [17]. They found the BLSs in some parameter regions, and the glial cell serves an important function in the appearance of BLSs. However, compared with our model, the glial cell in Ref. [16], [17] modulates neuronal behaviour behavior by removing excess potassium from the extracellular space. Postnov *et* *al.* have found the postsynaptic neuron response to the presynaptic neuron by bursting-like firing in their modeling work regarding the effect of glial cell, but the presynaptic neuron fires successively [37]. This finding differs from our results because the BLSs always appear in N1 and N2 simultaneously. Theoretically, the resting membrane potential during the bursting spikes is attributed to the inhibitory effect of the astrocyte to N1. In Fig. 5, the time series of the calcium concentration in the astrocyte and total current in N1(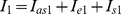) are depicted to correspond to Fig. 4. The calcium concentration is oscillating. When 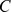 is larger than 196.69 nM, the increase of the inhibitory current 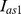
(negative) will cause 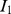 to decrease to a low level. Otherwise, when 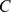 is larger than 196.69 nM, the inhibitory current vanishes, and 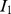 approaches 10 *µ*A/cm^2^. The red dashed lines in Fig. 5(b) and (d) represent 6.24 *µ*A/cm^2^. Obviously, while 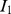 decreases to a value less than 6.24 *µ*A/cm^2^, the N1 will possess the resting membrane potential. In Fig. 5(b), although 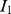 decreases owing to the larger 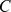, 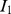 is always larger than 6.24 *µ*A/cm^2^. As a result, the successive firing of N1 will not be stopped. In Fig. 5 (d), 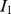 decreases to values less than 6.24 *µ*A/cm^2^ periodically. When 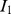 is less than 6.24 *µ*A/cm^2^, the N1 possesses the resting membrane potential, and BLSs are produced. Then, the BLSs are transferred to N2 through the excitatory synapse.

**Figure 6:**
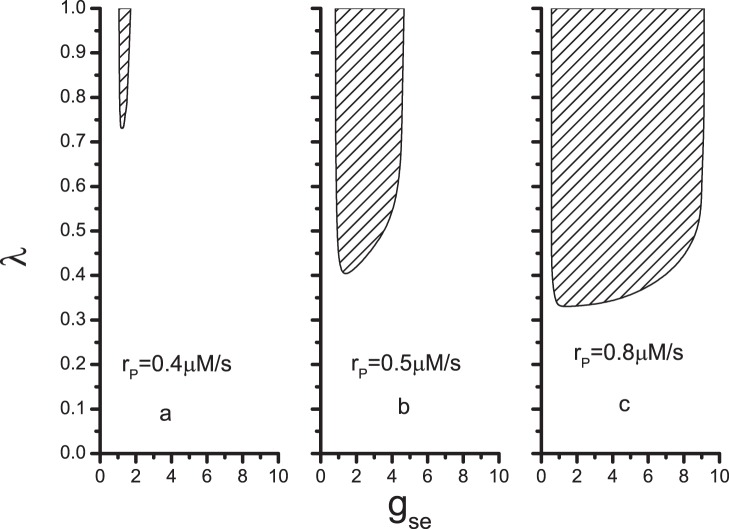
Parameter region in which BLSs are produced. The values of parameters 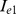 and 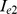 are same as in Fig. 2. The value of 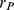 equals to (a) 0.4 *µ*M/s; (b) 0.5 *µ*M/s; (c) 0.8 *µ*M/s.

The effect of astrocyte serves an important function in the production of the BLSs. As previously mentioned, the excitatory coupling strength determines the the information transmission from N1 to N2 significantly. Thus, we will identify the region of parameter 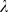 and 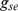, in which the BLSs are produced. Additionally, the IP_3_ production rate 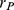 has been proven to be associated with the expression level of mGluRs in astrocytes. The enhanced production of IP_3_ corresponds to over-expressed mGluRs. Over-expression of mGluRs has been reported to facilitate the seizure-like oscillations in the neurons [26]. In our study, three typical values of 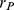, 0.4, 0.5 and 0.8 *µ*M/s, are selected to represent the normal, intermediate, and enhanced expression level of mGluRs, respectively. The shadow regions in Fig. 6 are the parameter regions in which the BLSs can be found. First, the BLSs appear for an intermediate value of 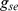. Extremely large or small 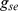 both make the calcium concentration approach a steady value less than 196.69 nM. In our model, the astrocyte fails to feedback to the neurons by 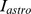 when 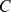 is less than 196.69 nM. Thus, the BLSs are not produced. Second, only if 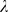 is larger than a critical value do BLSs appear. Thus, we can conclude that the existence of astrocyte is an important condition for the production of the BLSs. Finally, the area of the shadow regions decreases sharply with decreasing 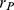. Enhanced expression level of mGluRs favors the BLSs. Although the models are different in previous literatures, the similar results have been obtained that the calcium dynamics in the astrocyte strongly affect the neural activity [5], [15].

**Figure 7:**
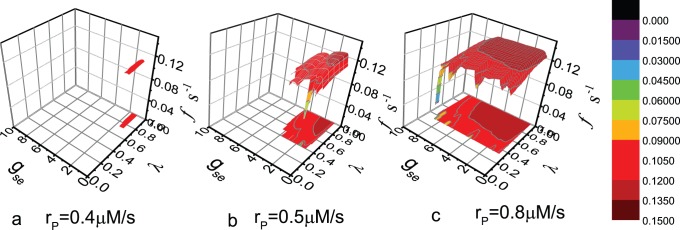
Rate of occurrence of the BLSs vs. the values of parameters 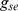 and 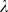. The values of parameters 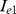, 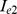, and 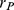 are same as in Fig. 6.

The rate of occurrence of the BLSs is then calculated. In Fig. 7, the rate 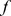 is approximately 0.12 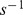 and is not very sensitive to the parameters, once the values of the parameters are within the shadow regions in Fig. 6. More accurately, 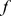 is maximum, and remains constant in the center of the shadow regions. 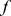 decreases when the parameter values change from the center to the edge of the regions. Furthermore, 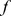 increases with the enhancement of the expression level of mGluRs. Although Cressman Jr. *et* *al.* have not investigated the effect of astrocyte on the rate of the BLSs clearly, Ref. [16] shows that the rate increases with the enhancement of glial strength, and the rate is at the scale from 0.01 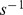 to 0.1 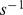. This finding is in accordance with our results qualitatively.

### Time Delay and Information Distortion

**Figure 8:**
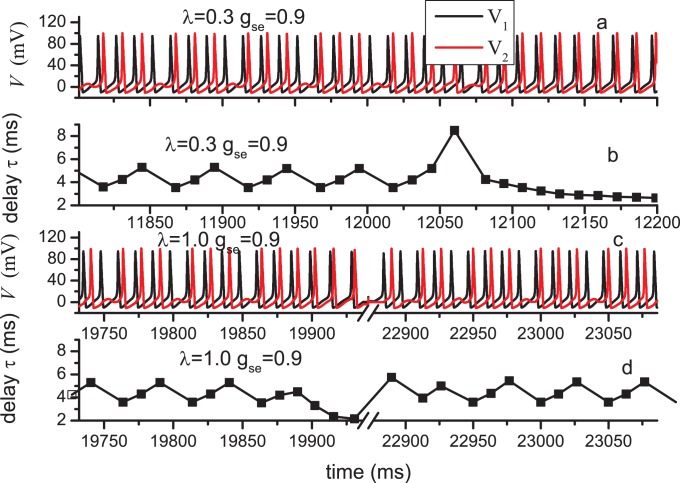
Time series of membrane potential in N1, N2 and corresponding time delay *τ* for different parameter values. (a)(b) 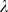 = 0.3, 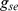 = 0.9; (c)(d) 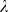 = 1.0, 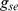 = 0.9. Note that in the broken regions of (c)and (d), the membrane potential remain on the resting states.

Synaptic transmission is widely accepted to involve time delay attributed to the signal propagation time [47]. Theoretically, neuronal models with time delay have received considerable attention. Delay-induced coherent oscillation [48] is found in neuronal network as well as in other coupled systems. Delay-enhanced synchronization [23], [49] may be relevant for neuronal networks to establish a concept of collective information processing in the presence of delayed information transmission. Our recent works find that delay cooperating with diversity can induce fruitful synchronization transitions [22]. Herein, the delay in the information transmission between two neurons will be verified in the presence of of astrocytes. As an example, in Fig. 8, the time series of 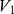 and 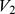 are recorded to show delay in the information transmission from N1 to N2. The spiking times in N2 always lag behind that in N1. The time delay 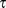 is the time interval between two closest spikes in the two neurons. No matter whether the BLSs are produced or not, the time delay does exist in the information transmission. This time delay are also found in the previous modelling work studying the effect of astrocytes in neuron system [40]. Fig. 8 (b) and (d) show the time delay 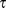 corresponding to (a) and (c), respectively. 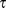 is not constant but oscillates irregularly. However, the oscillatory amplitude is not large; 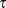 possesses low-amplitude changing around a average value.

**Figure 9:**
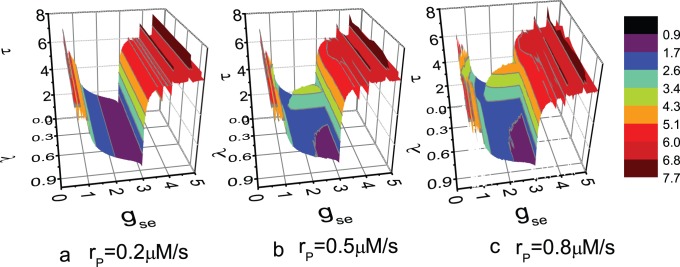
Average *τ* vs. the values of parameters 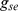 and 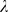. The value of 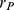 equals to (a) 0.2 *µ*M/s; (b) 0.5 *µ*M/s; (c) 0.8 *µ*M/s.

The average value of 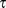 is calculated for different parameter values. Fig. 9 shows that with increasing 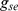, 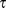 decreases to a minimum first and then increases to a saturated value. The decrease of 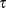 for small 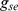 corresponds to the increase of synchronization in Ref. [40]. The intermediate value of 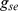 corresponding to the minimum 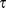 is about 2.96, and this value is independent of the expression level of mGluRs. Furthermore, for low expression level of mGluRs (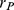 = 0.2), 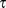 is totally independent on the value of 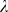. With increasing 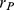, the minimal 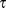 will be influenced by 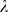. Fig. 9(b) and (c) show that the minimal 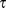 reaches a maximum for an intermediate value of 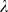, which is similar to the phenomenon of resonance found in random systems.

**Figure 10:**
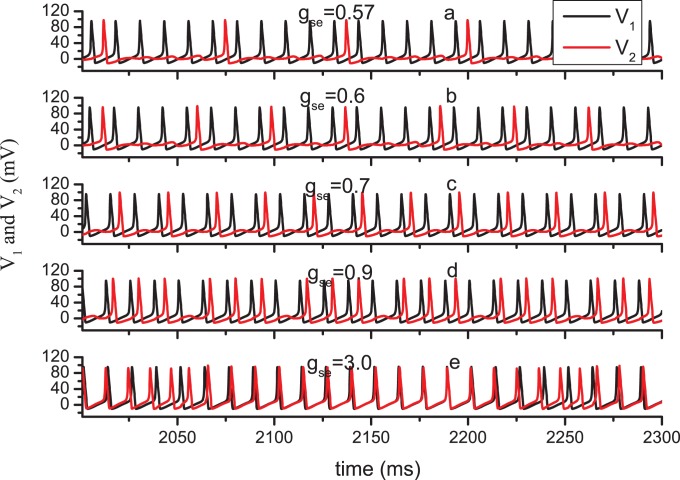
Time series of membrane potential in N1 and N2 illustrating the missing spikes. The parameter value 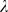 = 0.5, 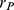 = 0.8 *µ*M/s. The value of 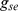 equals to (a) 0.57; (b)0.6; (c) 0.7; (d) 0.9; (e) 3.0.

Then, we will turn to another interesting phenomenon implied in Fig. 2 and 8. To exhibit this phenomenon clearly, the time series of 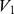 and 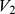 for different parameter values are depicted in Fig. 10. Notably, N2 does not respond to every spike in N1 through a corresponding spike accurately, i.e., large amounts of spikes are “missed” during the transmission from N1 to N2. Generally, the neuronal information is deemed to be coded in the spike timing or rate. Thus, the missing of spikes may relate to the distortion of the information transmission. Herein, we define the distortion ratio 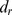 by the ratio between the spike number in N1 and N2. Obviously, all the spikes in N1 respond by spiking in N2 for sufficient coupling strength. If 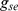 is reduced, the number of missing spikes increases, i.e., 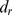 increases. In Fig. 10, the values of the parameter 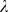 and 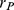 are set as 0.5 and 0.8, respectively. For these parameter values, the BLSs are produced. In fact, the calculation shows that this kind of missing spike may occur whether the BLSs are produced or not.

**Figure 11:**
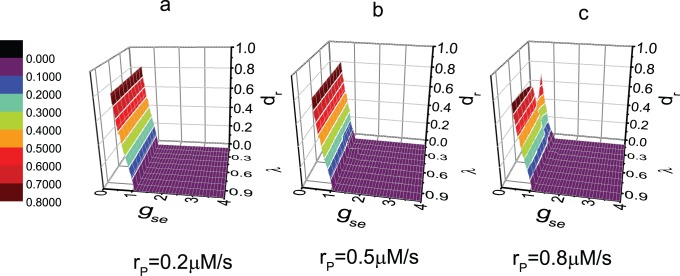
Distortion ratio 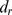 vs. the values of parameters 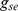 and 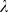. The value of 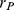 equals to (a) 0.2 *µ*M/s; (b) 0.5 *µ*M/s; (c) 0.8 *µ*M/s.

**Figure 12:**
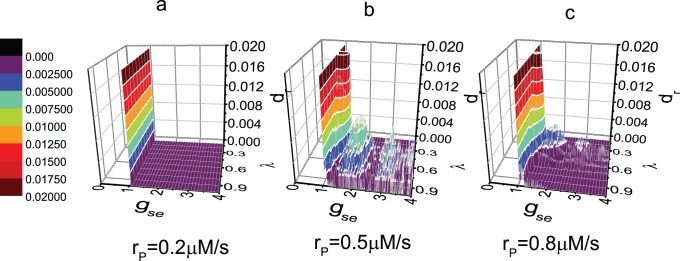
Distortion ratio 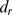 vs. the values of parameters 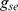 and 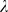. The value of 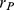 equals to (a)0.2 *µ*M/s; (b) 0.5 *µ*M/s; (c) 0.8 *µ*M/s. Note that the scale of 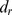 axis differs from that in Fig. 11.

The distortion rate 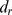 is calculated for different parameter values. Fig. 11 shows that the missing spike occurs mainly for small coupling strength 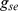. 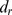 decreases to zero sharply if 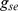 is increased from 0.56. The critical value of 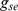, for which 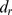 decreases to zero, is about 1.06. Comparing the three figures in Fig. 11, 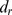 is almost independent of 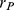 and 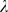. Even the critical value of 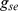 1.06 does not change with the changing of 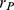 and 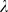. Thus, we can conclude that the effect of astrocyte does not serve an important function in the occurrence of missing spike. To exhibit the slight effect of astrocyte, the values of 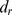 are amplified in Fig. 12. For intermediate or enhanced expression level of mGluRs, 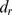 is non-zero with an intermediate value of 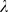, whereas 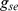 is larger than the critical value 1.06. We conclude that the effect of astrocyte induces the occurrence of miss of very few spike. Obviously, the accidental miss of spike does not result in the distortion of the information.

## Conclusions

In this paper, the information transmission between neurons is studied by using a model that contains two neurons and one astrocyte. First, we identify the parameter region in which the information can be transferred from N1 to N2. The effect of astrocyte does not influence this parameter region. Secondly, in the parameter region for information transmission, we find BLSs in two neurons simultaneously. The parameter values for the occurrence of BLSs are also identified, and the results show that the higher expression level of mGluRs and the existence of astrocyte facilitate the occurrence of BLSs. Meanwhile, the rate for the occurrence of BLSs is calculated, and the rate is not very sensitive to the parameters. Third, time delay in information transmission is studied. The results show that 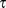 is not constant but oscillate with small amplitude. The average value of 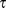 is dependent on 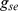 sensitively, but almost independent of 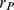 and 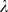. Finally, we found amounts of spikes are “missed” during the transmission from N1 to N2. This distortion occurs mainly for small coupling strength 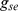. Although the astrocyte also induces very few missing spikes, it does not result in the distortion of the information.

Although glial cells have been widely accepted to serve an important function in synaptic transmission in neuron system, theoretical knowledge on the mechanism of interaction between glial cell and neurons is lacking. The modelling studies in this paper can help us to understand the mechanism by which the astrocytes participate in neuronal information transmission.

## References

[1] Volterra A, Meldolesi J (2005) Astrocytes, from brain glue to communication elements: the revolution continues. Nature Rev Neurosci 6: 626–640. 16025096 10.1038/nrn1722

[2] Giaume C, Koulakoff A, Roux L, Holcman D, Rouach N (2010) Astroglial networks: a step further in neuroglial and gliovascular interactions. Nature Rev Neurosci 11: 87–99. 20087359 10.1038/nrn2757

[3] Auld D S, Robitaille R (2003) Glial cells and neurotransmission: an inclusive view of synaptic function. Neuron 40: 389–400. 14556716 10.1016/s0896-6273(03)00607-x

[4] Halassa MM, Haydon PG (2010) Integrated brain circuits: astrocytic networks modulate neuronal activity and behavior. Annu Rev Physiol 72: 335–355. 20148679 10.1146/annurev-physiol-021909-135843 PMC3117429

[5] Postnov DE, Ryazanova LS, Sosnovtseva OV (2007) Functional modeling of neuralCglial interaction. BioSystems 89: 84–91. 17320272 10.1016/j.biosystems.2006.04.012

[6] Jourdain P, Bergersen LH, Bhaukaurally K, Bezzi P, Santello M, et al (2007) Glutamate exocytosis from astrocytes controls synaptic strength. Nature Neuosci 10: 331–339. 10.1038/nn1849 17310248

[7] Fellin T, Pascual O, Gobbo S, Pozzan T, Haydon PG, et al (2004) Neuronal Synchrony Mediated by Astrocytic Glutamate through Activation of Extrasynaptic NMDA Receptors. Neuron 43: 729–743. 15339653 10.1016/j.neuron.2004.08.011

[8] Perea G, Araque A (2005) Properties of Synaptically Evoked Astrocyte Calcium Signal Reveal Synaptic Information Processing by Astrocytes. J Neuosci 25(9): 2192–2203. 10.1523/JNEUROSCI.3965-04.2005 PMC6726085 15745945

[9] Auld DS, Robitaille R (2003) Glial Cells and Neurotransmission: An Inclusive View of Synaptic Function. Neuron 40: 389–400. 14556716 10.1016/s0896-6273(03)00607-x

[10] Wang Z, Haydon PG, Yeung ES (2000) Direct observation of calcium-independent intercellular ATP signalling in astrocytes. Anal Chem 72(9): 2001–2007. 10815957 10.1021/ac9912146

[11] Guthrie PB, Knappenberger J, Segal M, Bennett MVL, Charles AC, et al (1999) ATP released from astrocytes mediates glial calcium waves. J Neurosci 19(2): 520–528. 9880572 10.1523/JNEUROSCI.19-02-00520.1999 PMC6782195

[12] Zhang JM, Wang HK, Ye CQ, Ge W, Chen Y, et al (2003) ATP released by astrocytes mediates glutamatergic activity-dependent heterosynaptic suppression. Neuron 40: 971–982. 14659095 10.1016/s0896-6273(03)00717-7

[13] Stamatakis M, Mantzaris NV (2006) Modeling of ATP−mediated signal transduction and wave propagation in astrocytic cellular networks. J Theor Biol 241: 649–668. 16460762 10.1016/j.jtbi.2006.01.002

[14] Garbo AD, Barbi M, Chillemi S, Alloisio S, Nobile M (2007) Calcium signalling in astrocytes and modulation of neural activity. Biosystems 89: 74–83. 17196325 10.1016/j.biosystems.2006.05.013

[15] Garbo AD (2009) Dynamics of a minimal neural model consisting of an astrocyte, a neuron, and an interneuron. J Biol Phys 35: 361–382. 19669428 10.1007/s10867-009-9143-2 PMC2750740

[16] Cressman JR Jr, Ullah G, Ziburkus J, Schiff SJ, Barreto E (2009) The influence of sodium and potassium dynamics on excitability, seizures, and the stability of persistent states: I. Single neuron dynamics. J Comput Neurosci 26: 159–170. 19169801 10.1007/s10827-008-0132-4 PMC2704057

[17] Ullah G, Cressman JR Jr, Barreto E, Schiff SJ (2009) The influence of sodium and potassium dynamics on excitability, seizures, and the stability of persistent states: II. Network and glial dynamics. J Comput Neurosci 26: 171–183. 19083088 10.1007/s10827-008-0130-6 PMC2951284

[18] Araque A, Parpura V, Sanzgiri RP, Haydon PG (1999) Tripartite synapses: glia, the unacknowledged partner. Trends Neurosci 22: 208–215. 10322493 10.1016/s0166-2236(98)01349-6

[19] Perea G, Navarrete M, Araque A (2009) Tripartite synapses: astrocytes process and control synaptic information. Trends Neurosci 32: 421–431. 19615761 10.1016/j.tins.2009.05.001

[20] Postnov DE, Ryazanova LS, Brazhe NA, Brazhe AR, Maximov GV, et al (2008) Giant Glial Cell: New Insight Through Mechanism-Based Modeling. J Biol Phys 34: 441–457. 19669488 10.1007/s10867-008-9070-7 PMC2585624

[21] Pannascha U, Vargovb L, Reingruber J, Ezana P, Holcmand D, et al (2011) Astroglial networks scale synaptic activity and plasticity. Proc. Natl. Acad. Sci. USA 108: 8467–8472. 10.1073/pnas.1016650108 PMC3100942 21536893

[22] Tang J, Ma J, Yi M, Xia H, Yang X (2011) Delay and diversity-induced synchronization transitions in a small-world neuronal network. Phy Rev E 83: 046207. 10.1103/PhysRevE.83.046207 21599270

[23] Wang Q, Perc M, Duan Z, Chen G (2009) Synchronization transitions on scale-free neuronal networks due to finite information transmission delays. Phy Rev E 80: 026206. 10.1103/PhysRevE.80.026206 19792230

[24] Gao Y, Wang JJ (2012) Doubly stochastic coherence in complex neuronal networks. Phy Rev E 86: 051914. 10.1103/PhysRevE.86.051914 23214821

[25] Perc M, Marhl M (2005) Amplification of information transfer in excitable systems that reside in a steady state near a bifurcation point to complex oscillatory behavior. Phy Rev E 71: 026229. 10.1103/PhysRevE.71.026229 15783409

[26] Nadkarni S, Jung P (2003) Spontaneous Oscillations of Dressed Neurons: A New Mechanism for Epilepsy? Phy Rev Lett 91: 268101. 10.1103/PhysRevLett.91.268101 14754091

[27] Parpura V, Haydon P (2000) Physiological astrocytic calcium levels stimulate glutamate release to modulate adjacent neurons. Proc Natl Acad Sci USA 97: 8629. 10900020 10.1073/pnas.97.15.8629 PMC26999

[28] Allegrini P, Fronzoni L, Pirino D (2009) The influence of the astrocyte field on neuronal dynamics and synchronization. J Biol Phys 35: 413–423. 19669414 10.1007/s10867-009-9166-8 PMC2750747

[29] Nadkarni S, Jung P (2004) Dressed neurons: modeling neural-glial Interactions. Phys Biol 1: 35–41. 16204820 10.1088/1478-3967/1/1/004

[30] Volman V, Bazhenov M, Sejnowski TJ (2012) Computational models of neuron-astrocyte interac-tion in epilepsy. Front Comput Neurosci 6: 58. 23060780 10.3389/fncom.2012.00058 PMC3459315

[31] Silchenko AN, Tass PA (2008) Computational modeling of paroxysmal depolarization shifts in neurons induced by the glutamate release from astrocytes. Biol Cybern 98: 61–74. 18064484 10.1007/s00422-007-0196-7

[32] Amiri M, Bahrami F, Janahmadi M (2011) Functional modeling of astrocytes in epilepsy: a feed-back system perspective. Neural Comput Applic 20: 1131–1139.

[33] Amiri M, Bahrami F, Janahmadi M (2012) On the role of astrocytes in epilepsy: A functional modeling approach. Neurosci Res 72: 172–180. 22138615 10.1016/j.neures.2011.11.006

[34] Amiri M, Bahrami F, Janahmadi M (2012) Modified thalamocortical model: A step towards more understanding of the functional contribution of astrocytes to epilepsy. J Comput Neurosci 33: 285–299. 22382677 10.1007/s10827-012-0386-8

[35] Tian GF, Azmi H, Takano T, Xu Q, Peng W, et al (2005) An astrocytic basis of epilepsy. Nat Med 11: 973–981. 16116433 10.1038/nm1277 PMC1850946

[36] Pereira A Jr, Furlan FA (2009) On the role of synchrony for neuron−astrocyte interactions and perceptual conscious processing. J Biol Phys 35: 465–481. 19669426 10.1007/s10867-009-9147-y PMC2750741

[37] Postnov DE, Koreshkov RN, Brazhe NA, Brazhe AR, Sosnovtseva OV (2009) Dynamical patterns of calcium signaling in a functional model of neuronCastrocyte networks. J Biol Phys 35: 425–445. 19669421 10.1007/s10867-009-9156-x PMC2750744

[38] Amiri M, Montaseri G, Bahrami F (2011) On the role of astrocytes in synchronization of two coupled neurons: a mathematical perspective. Biol Cybern 105: 153–166. 21935706 10.1007/s00422-011-0455-5

[39] Amiri M, Bahrami F, Janahmadi M (2012) Functional contributions of astrocytes in synchronization of a neuronal network model. J Theor Biol 292: 60–70. 21978738 10.1016/j.jtbi.2011.09.013

[40] Amiri M, Hosseinmardi N, Bahrami F, Janahmadi M (2013) Astrocyte−neuron interaction as a mechanism responsible for generation of neural synchrony: a study based on modeling and experiments. J Comput Neurosci 34: 489–504. 23661228 10.1007/s10827-012-0432-6

[41] Hodgkin AL, Huxley AF (1952) A quantitative description of membrane current and its application to conduction and excitation in nerve. J Physiol 117: 500–544. 12991237 10.1113/jphysiol.1952.sp004764 PMC1392413

[42] Terman D, Rubin JE, Yew AC, Wilson CJ (2002) Activity patterns in a model for the subtha- lamopallidal network of the basal ganglia. J Neurosci 22: 2963–2976. 11923461 10.1523/JNEUROSCI.22-07-02963.2002 PMC6758326

[43] Li YX, Rinzel J (1994) Equations for InsP3 Receptor-mediated [Ca2+]*i* Oscillations Derived from a Detailed Kinetic Model: A Hodgkin−Huxley Like Formalism. J Theor Biol 166: 461–473. 8176949 10.1006/jtbi.1994.1041

[44] Bowser DN, Khakh BS (2004) ATP excites interneurons and astrocytes to increase synaptic inhibition in neuronal networks. J Neurosci 24: 8606–8620. 15456834 10.1523/JNEUROSCI.2660-04.2004 PMC6729897

[45] Fellin T, Pascual O, Haydon PG (2006) Astrocytes coordinate synaptic networks: balanced excitation and inhibition. J Physiol 21: 208–215. 10.1152/physiol.00161.2005 16714479

[46] Koizumi S, Fujishita K, Tsuda M, Shigemoto−Mogami Y, Inoue K (2003) Dynamic inhibition of excitatory synaptic transmission by astrocyte−derived ATP in hyppocampal cultures. Proc Natl Acad Sci USA 100: 11023–11028. 12958212 10.1073/pnas.1834448100 PMC196920

[47] Kandel ER, Schwartz JH, Jessell TM (1991) Principles of Neural Science. Elsevier Amsterdam.

[48] Wang Q, Perc M, Duan Z, Chen G (2008) Delay−enhanced coherence of spiral waves in noisy Hodgkin−Huxley neuronal networks. Phys. Lett. A 372: 5681.

[49] Gerstner W (1996) Rapid Phase Locking in Systems of Pulse-Coupled Oscillators with Delays. Phys Rev Lett 76: 1755. 10060509 10.1103/PhysRevLett.76.1755

