# Correction: Information Transmission in a Neuron-Astrocyte Coupled Model

**DOI:** 10.1371/annotation/4bb2348f-d05c-499b-80b8-58cbb0d038a0

**Published:** 2014-01-23

**Authors:** Jun Tang, Jin-Ming Luo, Jun Ma

Errors were introduced during the production process. Text, including Equations 6-7, was moved from the main body of the article into the legend of Figure 5. This article was republished on January 17th, 2014, in order to correct these errors. The publisher apologizes for these errors. You can view the original version of the article here: http://www.plosone.org/corrections/pone.0080324.001.cn.pdf

